# Hyperpolarized [1-^13^C]-pyruvate MRS evaluates immune potential and predicts response to radiotherapy in cervical cancer

**DOI:** 10.1186/s41747-024-00445-1

**Published:** 2024-04-10

**Authors:** Gigin Lin, Ching-Yi Hsieh, Ying-Chieh Lai, Chun-Chieh Wang, Yenpo Lin, Kuan-Ying Lu, Wen-Yen Chai, Albert P. Chen, Tzu-Chen Yen, Shu-Hang Ng, Chyong-Huey Lai

**Affiliations:** 1 Department of Medical Imaging and Intervention, Chang Gung Memorial Hospital at Linkou, 5 Fuhsing St, Guishan, 33382 Taoyuan Taiwan; 2 Department of Medical Imaging and Radiological Sciences, Chang Gung University, Taoyuan, Taiwan; 3 Clinical Metabolomics Core Laboratory, Chang Gung Memorial Hospital at Linkou, Taoyuan, Taiwan; 4 Research Center for Radiation Medicine, Chang Gung University, Taoyuan, Taiwan; 5 Department of Radiation Oncology, Chang Gung Memorial Hospital at Linkou, Taoyuan, Taiwan; 6 GE Healthcare, Toronto, Canada; 7 Department of Nuclear Medicine and Molecular Imaging Center, Chang Gung Memorial Hospital, Chang Gung University College of Medicine, Taoyuan, Taiwan; 8 Division of Gynecologic Oncology, Gynecologic Oncology Research Center, Chang Gung Memorial Hospital and Chang Gung University College of Medicine, Taoyuan, Taiwan

**Keywords:** Carbon-13, Magnetic resonance spectroscopy, Radiotherapy, Spleen, Uterine cervical neoplasms

## Abstract

**Background:**

Monitoring pyruvate metabolism in the spleen is important for assessing immune activity and achieving successful radiotherapy for cervical cancer due to the significance of the abscopal effect. We aimed to explore the feasibility of utilizing hyperpolarized (HP) [1-^13^C]-pyruvate magnetic resonance imaging (MRI) and magnetic resonance spectroscopy (MRS) to evaluate pyruvate metabolism in the human spleen, with the aim of identifying potential candidates for radiotherapy in cervical cancer.

**Methods:**

This prospective study recruited six female patients with cervical cancer (median age 55 years; range 39–60) evaluated using HP [1-^13^C]-pyruvate MRI/MRS at baseline and 2 weeks after radiotherapy. Proton (^1^H) diffusion-weighted MRI was performed in parallel to estimate splenic cellularity. The primary outcome was defined as tumor response to radiotherapy. The Student *t*-test was used for comparing ^13^C data between the groups.

**Results:**

The splenic HP [1-^13^C]-lactate-to-total carbon (tC) ratio was 5.6-fold lower in the responders than in the non-responders at baseline (*p* = 0.009). The splenic [1-^13^C]-lactate-to-tC ratio revealed a 1.7-fold increase (*p* = 0.415) and the splenic [1-^13^C]-alanine-to-tC ratio revealed a 1.8-fold increase after radiotherapy (*p* = 0.482). The blood leukocyte differential count revealed an increased proportion of neutrophils two weeks following treatment, indicating enhanced immune activity (*p* = 0.013). The splenic apparent diffusion coefficient values between the groups were not significantly different.

**Conclusions:**

This exploratory study revealed the feasibility of HP [1-^13^C]-pyruvate MRS of the spleen for evaluating baseline immune potential, which was associated with clinical outcomes of cervical cancer after radiotherapy.

**Trial registration:**

ClinicalTrials.gov NCT04951921, registered 7 July 2021.

**Relevance statement:**

This prospective study revealed the feasibility of using HP ^13^C MRI/MRS for assessing pyruvate metabolism of the spleen to evaluate the patients’ immune potential that is associated with radiotherapeutic clinical outcomes in cervical cancer.

**Key points:**

• Effective radiotherapy induces abscopal effect via altering immune metabolism.

• Hyperpolarized ^13^C MRS evaluates patients’ immune potential non-invasively.

• Pyruvate-to-lactate conversion in the spleen is elevated following radiotherapy.

**Graphical Abstract:**

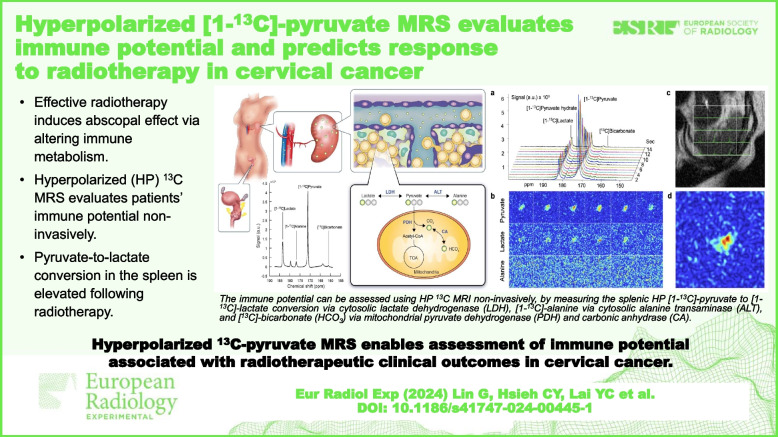

**Supplementary Information:**

The online version contains supplementary material available at 10.1186/s41747-024-00445-1.

## Background

Concurrent chemoradiotherapy stands as the established approach for treating locally advanced cervical cancer at International Federation of Gynecology and Obstetrics (FIGO) stage IB2–IVA. Nevertheless, the 5-year survival rates for such cases remain modest [1], particularly when there are sizable tumor volumes, indicating a less favorable prognosis [2]. Radiation therapy prompts the release of tumor-associated antigens and damaged DNA, potentially triggering the abscopal effect—a systemic antitumor response involving immunological activation [3]. The Warburg effect, previously established in cancer biology, represents a common characteristic among actively proliferating cells. Aerobic glycolysis favoring the conversion of pyruvate to lactate is the principal pathway of glucose metabolism during lymphocyte activation. Governing energy metabolism may offer a means for T cells to transition reversibly between dormant and highly proliferative states [4]. Monitoring this phenomenon holds critical importance in assessing immune activity and the efficacy of radiotherapy [5]. Metabolic reprogramming linked to lymphocyte activation predominantly manifests in the spleen [6]. However, the invasive nature and associated risk of bleeding deter the biopsy of the spleen [7]. As a result, there exists an unaddressed clinical necessity for a non-invasive imaging tool to gauge the immune competence of the spleen.

Hyperpolarized (HP) carbon-13 (^13^C)-magnetic resonance imaging (MRI) is an emerging molecular imaging technique that enables rapid and pathway-specific exploration of dynamic metabolic and physiologic processes that were previously difficult to image [8]. The first-in-human study proved the feasibility and safety of HP [1-^13^C]-pyruvate in non-invasively assessing tumor metabolism in prostate cancer [9]. Since then, further research has expanded the use of this technique to monitor metabolism in breast cancer [10], renal cell carcinoma [11], human skeletal muscle [12], and myocardium [13].

**Fig. 1:**
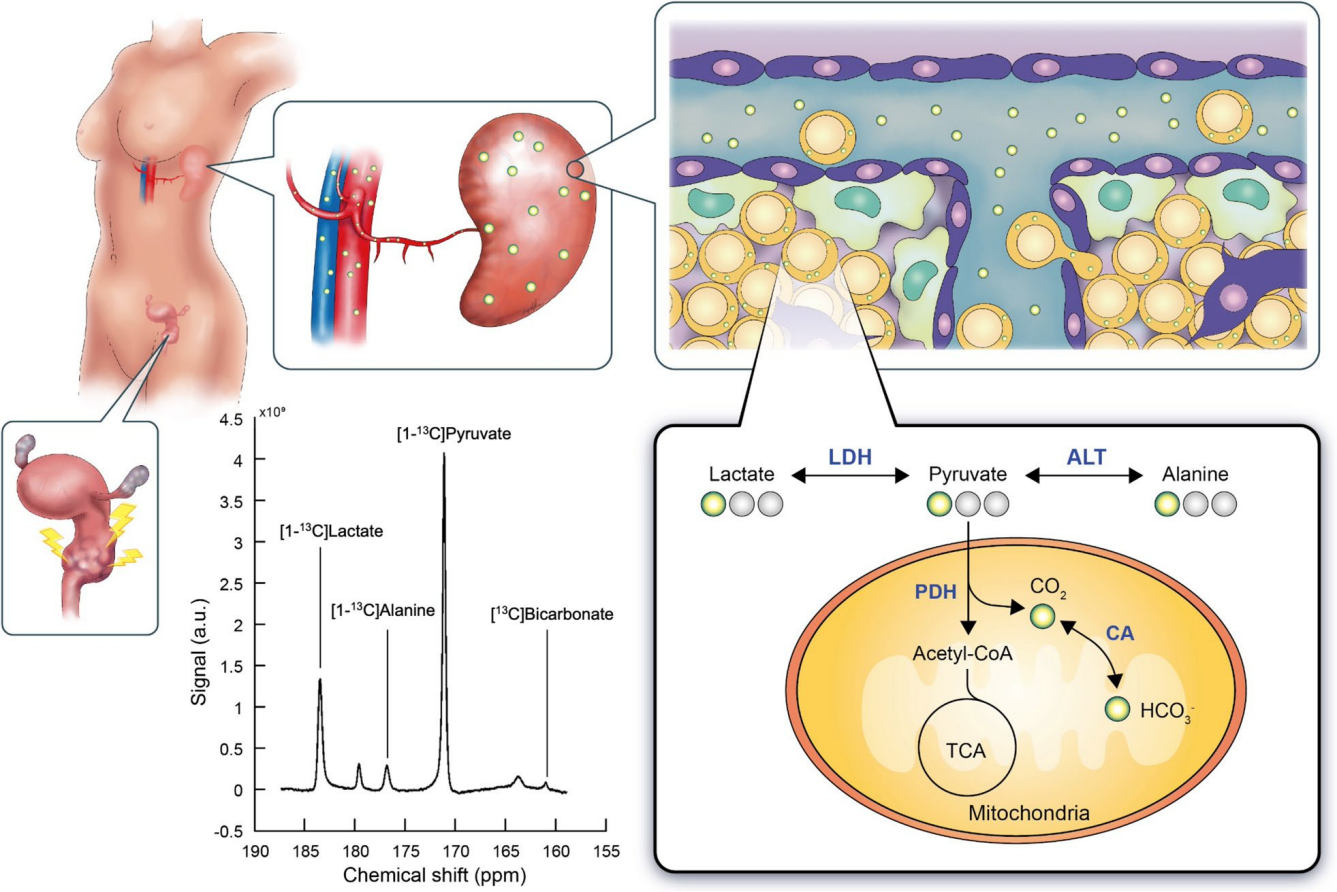
Study overview. Radiation therapy induces the abscopal effect that triggers metabolic alterations in immune system. Intravenously administration of hyperpolarized (HP) [1-^13^C]-pyruvate is rapidly distributed to spleen via arterial inflow, entering the interstitial space and being transported into the intracellular space. The pyruvate metabolism in the human spleen can be probed using magnetic resonance spectroscopy non-invasively, by measuring the HP [1-^13^C]-pyruvate to [1-^13^C]-lactate conversion via cytosolic lactate dehydrogenase (LDH), [1-^13^C]-alanine via cytosolic alanine transaminase (ALT), and [.^13^C]-bicarbonate (HCO_3_) via mitochondrial pyruvate dehydrogenase (PDH) and carbonic anhydrase (CA)

Intravenously injected HP [1-^13^C]-pyruvate is rapidly distributed to organs via arterial inflow, entering the interstitial space and being transported into the intracellular space, which accounts for around 70% of the splenic volume. A previous preclinical study using HP [1-13C]-pyruvate MRI has demonstrated that, following radiation exposure, there was a significant decrease in the pyruvate-to-lactate conversion rates observed in the cancer cells, while an increase was noted in the immune cells [14]. This study sheds light on the potential clinical applications of non-invasive HP MRI technique to monitor the immune status during radiotherapy. We hypothesize that HP [1-^13^C]-pyruvate magnetic resonance spectroscopy (MRS) of the spleen could capture the intracellular pyruvate metabolism flux of the immune system, as shown in Fig. 1.

The objective of this study was to explore the feasibility of utilizing HP [1-^13^C]-pyruvate MRS to evaluate pyruvate metabolism in the human spleen, with the aim of identifying potential candidates for radiotherapy in cervical cancer.

## Methods

### Study protocol and clinical screening of patients

This prospective study was approved by the local institutional review board (IRB 201702080A0, ClinicalTrials.gov ID: NCT04951921), compliant with the Health Insurance Portability and Accountability Act, and was conducted from July 2021 to May 2022 under approval by the Taiwanese Food and Drug Administration (investigational new drug application No. 110IND2017). Inclusion criteria were as follows: (1) confirmed diagnosis of cervical cancer via histological examination; (2) age of 20 years or older; (3) clinical stage of IB2-IV based on the International Federation of Gynecology and Obstetrics (FIGO) criteria; (4) a tumor size of at least 4 cm, confirmed by MRI or computed tomography; and (5) scheduled for curative non-surgical treatment. Exclusion criteria were as follows: (1) inability to undergo MRI due to contraindications such as claustrophobia; presence of a cardiac pacemaker, or metal implants in the pelvis; (2) inadequate function of the liver, kidney, or bone marrow; (3) presence of uncontrolled intercurrent illness including but not limited to active infection, symptomatic congestive heart failure, unstable angina pectoris, cardiac arrhythmia, or psychiatric illness/social situations that may interfere with compliance to study requirements; (4) pregnant or lactating women; and (5) known lymphoma or focal splenic lesion. HP [1-^13^C]-pyruvate MRI/MRS of the spleen was scheduled at baseline and 2 weeks after radiotherapy for the six female patients (median age 55, range 39–60 years) (Table 1). The patients had their first routine clinical follow-up 3 months after radiotherapy completion. Patients were classified as responders if they showed complete tumor remission, while non-responders were defined as patients with incomplete regression or progression as determined by MRI and/or 2-deoxy-2-[^18^F]-fluorodeoxyglucose (FDG) positron-emission/computed tomography (PET/CT).

**Table 1:** Demographic information of study participants

Variable	Data
Females (number)	6
Age (years)	55 (39–60)
Height (cm)	153 (139–167)
Weight (kg)	57 (40–85)
Body mass index (kg/m^2^)	24 (21–30)
Histopathology, squamous cell carcinoma (number)	6
Tumor size (mm)	45 (28–67)
Stage (number)	
T2b	4
T3	1
T4	1
N0	1
N1	4
N2	1
M0	5
M1	1

Data are absolute numbers or medians with their range in parentheses

### MRI methods

**Fig. 2:**
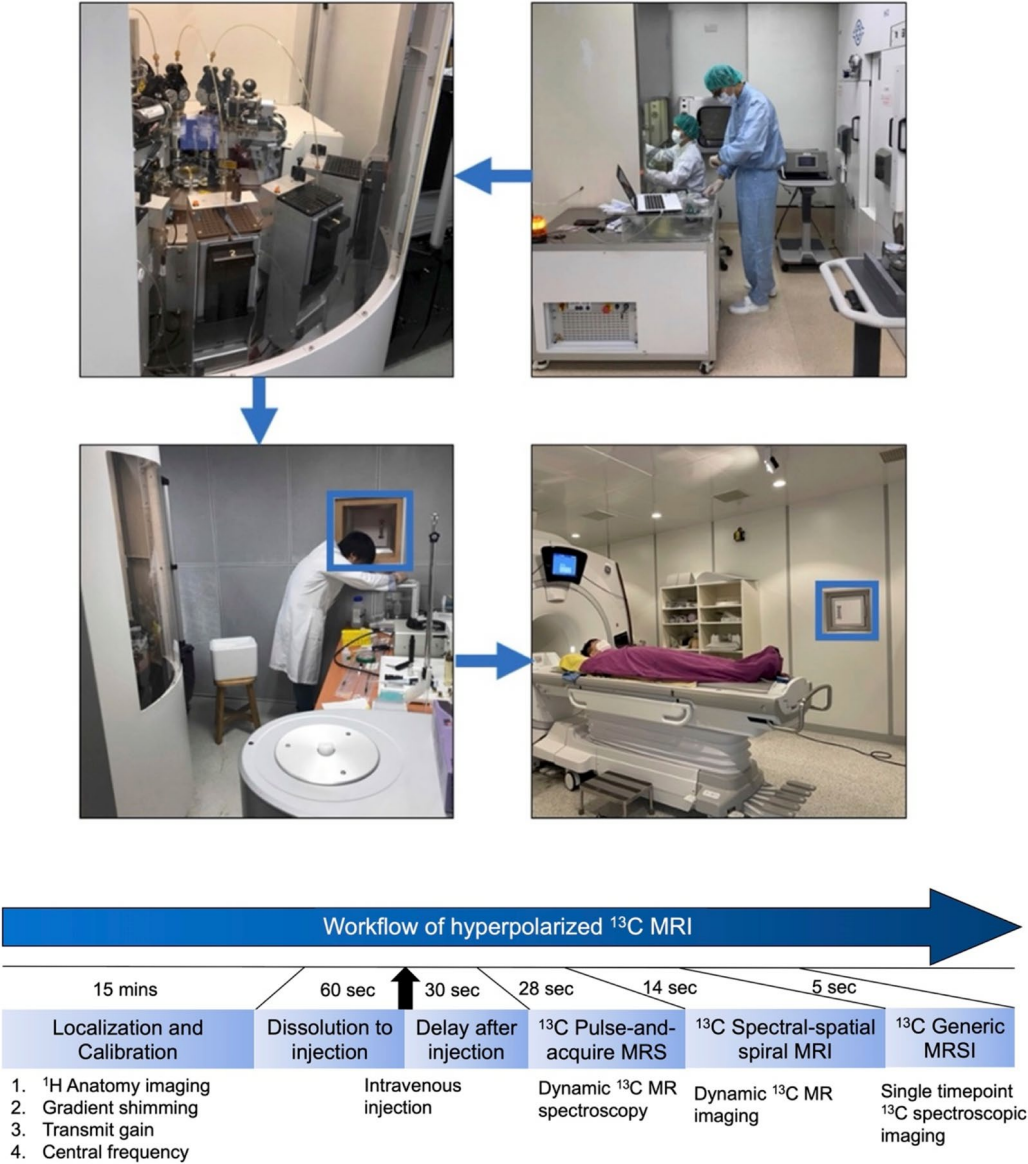
Hyperpolarized MR study protocol. The doses were prepared in a sterile environment and polarized on the same day as the patient’s visit. After the magnetic resonance scanning field was calibrated and localized, the hyperpolarized (HP) [1-^13^C]-pyruvate was dissolved and transferred into the scanner via a window (blue box) and administered to the patient intravenously. Dynamic ^13^C magnetic resonance spectroscopy (MRS) was performed 30 s after injection to track the delivery and metabolism of HP [1-^13^C]-pyruvate in the spleen. This was followed by dynamic ^13^C magnetic resonance imaging (MRI) and a single-timepoint ^13^C spectroscopic imaging (MRSI) to confirm the origin of HP ^13^C signals

**Fig. 3:**
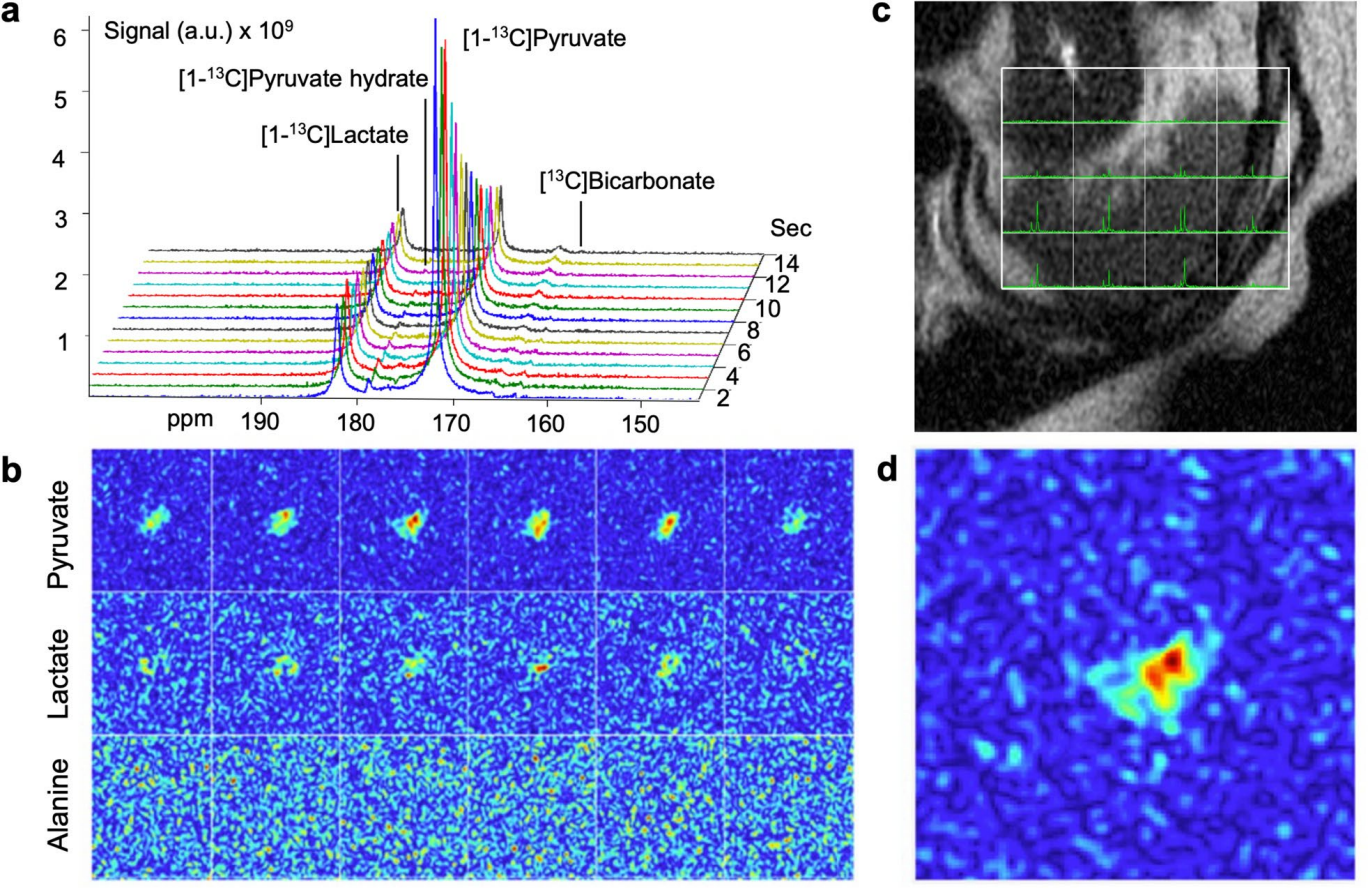
Dynamic ^13^C magnetic resonance (MR) spectroscopy and imaging of the spleen. Dynamic ^13^C MR spectroscopy of the spleen demonstrated the signals from HP [1-^13^C]-pyruvate, [1-^13^C]-lactate, [1-^13^C]-alanine, and [^13^C]-bicarbonate (**a**). Dynamic ^13^C MR imaging (**b**) and a single-timepoint ^13^C spectroscopic imaging (**c**) confirmed the origin of HP ^13^C signals from the spleen (**d**), corresponding to the anatomical ^1^H image in (**b**)

The study protocol is summarized in Fig. 2. All MRI/MRS studies were performed using a 3-T scanner (Discovery MR750w; GE Healthcare) with a 35-mm diameter ^13^C/^1^H multinuclear transmit–receive coil (RAPID Biomedical, Rimpar, Germany) for imaging the spleen in the supine position. A dose of 0.43 mL/kg of HP [1-^13^C]-pyruvate with a 250-mM concentration was given at 5 mL/s using a power injector (MedRad, Bayer Healthcare, Warrendale, USA). The study employed a slice-selective pulse-and-acquire sequence to acquire time-resolved free-induction-decay data for ^13^C MRS, which included the entire signal from the selected slice. In order to confirm the origin of HP ^13^C signals arising from the location of the spleen, a metabolite-selective multi-echo spiral imaging sequence was used to capture images of [1-^13^C]-pyruvate, [1-^13^C]-lactate, [1-^13^C]-alanine, and [^13^C]-bicarbonate in an interleaved manner (Fig. 3). The spectral-spatial radiofrequency pulse was used to selectively excite the labeled metabolites [15] followed by single shot spiral readout for each resonance. The ^13^C images were obtained when the product HP signals has reached its temporal maximum (*i.e.,* 30 s from the start of pyruvate injection) to maximize the signal-to-noise ratio of products. The blood and urine levels of pyruvate, lactate, and alanine at the time of imaging were analyzed by using a 600 MHz ^1^H nuclear magnetic resonance spectrometer. Further information regarding the protocol and data analysis can be found in the Supplementary material, which is available online.

### Statistical analysis

All data analyses were performed using Excel (version 16.70, Microsoft, Redmond, USA). Data are reported as the mean ± standard error, as standard error accounts for sample size, providing a more accurate estimate of the variability in the sample mean and offering a more reliable representation of the population parameter. Significant differences were evaluated using two-tailed Student *t*-test, based on the data distribution being approximately normal and that the population variance reasonably homogeneous. A paired *t*-test was used to compare data before and after radiotherapy, and an unpaired *t*-test was used to compare results between responders and non-responders. A *p*-value of < 0.05 indicated statistically significant.

## Results

**Fig. 4:**
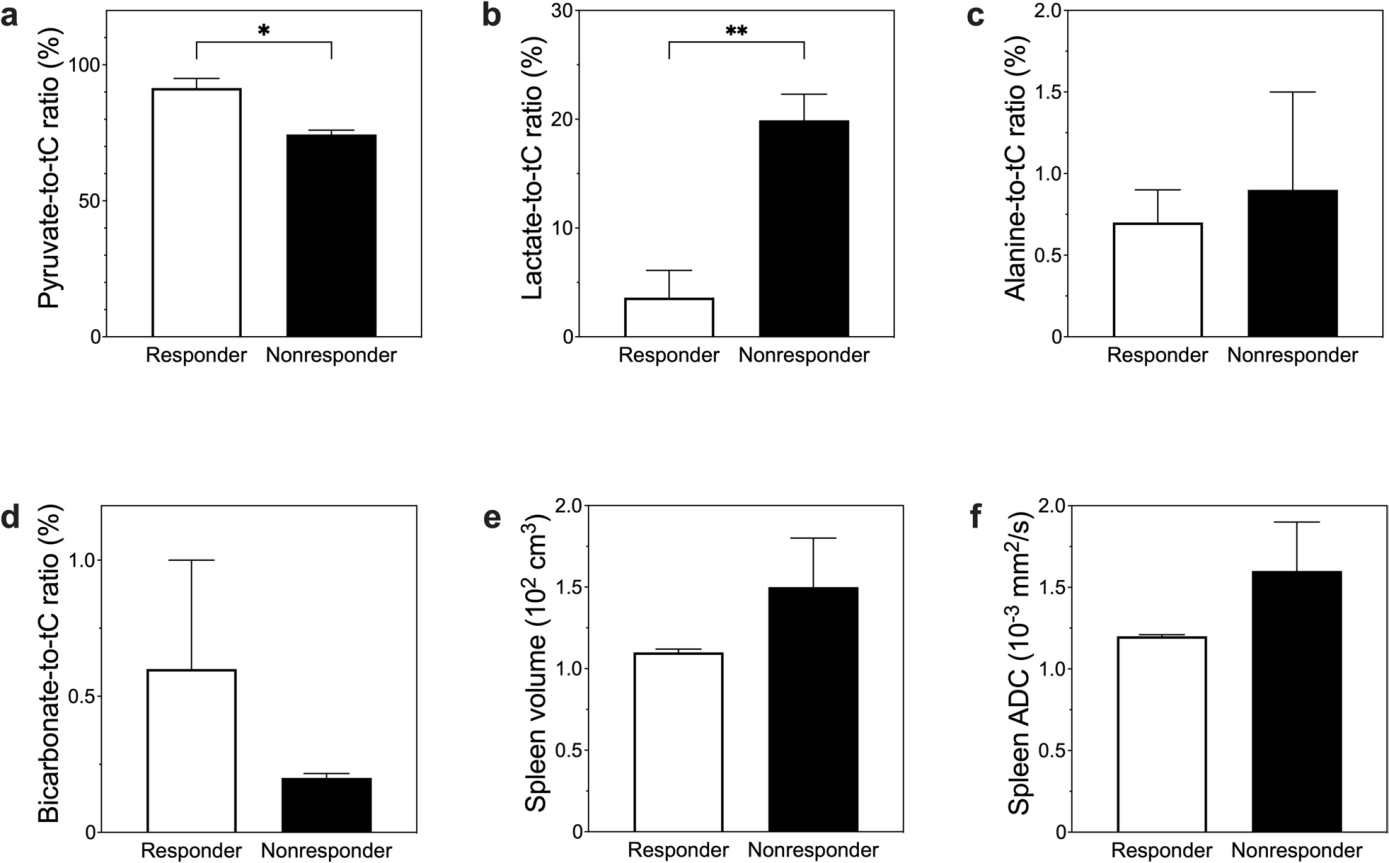
Hyperpolarized magnetic resonance (MR) spectroscopy and imaging of the spleen between responder *versus* non-responder. The baseline splenic hyperpolarized (HP) [1-^13^C]-pyruvate-to-tC ratio (%) was found to be significantly higher (**a**), whereas the HP [1-^13^C]-lactate-to-total carbon (tC) ratio (%) was 5.6-fold significantly lower (**b**), in responders compared to non-responders. There were no significant differences observed in the HP [1-^13^C]-alanine-to-tC ratio (**c**) and HP [.^13^C]-bicarbonate-to-tC ratio (**d**) as well as splenic volume (**e**) or apparent diffusion coefficient (ADC) values (**f**). *p < 0.05; **p < 0.01

HP [1-^13^C]-pyruvate, [1-^13^C]-lactate, [1-^13^C]-alanine, and [^13^C]-bicarbonate were detected in ^13^C spectra of the spleen, as shown in Fig. 1. Table 2 summarizes the baseline immune and metabolic characteristics between responders and non-responders. Based on MRS, the splenic [1-^13^C]-lactate-to-total carbon (tC) ratio (%) produced from HP [1-^13^C]-pyruvate was 5.6-fold lower in responders (mean ± standard error, 3.6 ± 2.5, *n* = 3) than in non-responders (mean, 19.9 ± 2.4; *n* = 3, *p* = 0.009). The HP [1-^13^C]-pyruvate-to-tC ratio (%) was higher in responders (mean, 91.5 ± 3.5, *n* = 3) than in non-responders (mean, 74.4 ± 1.6, *n* = 3, *p* = 0.011). The total HP ^13^C signal (tC), HP [1-^13^C]-alanine-to-tC ratio, and HP [^13^C]-bicarbonate-to-tC ratio showed no significant difference between the groups (Fig. 4). Two of the study cohort, both non-responders, also underwent baseline FDG PET/CT before treatment, with the spleen-to-liver standard uptake value being 1.19 and 1.00. Features of splenic morphology or cellularity did not show remarkable differences in the splenic volume or apparent diffusion coefficient (ADC) values. There were no significant differences between responders and non-responders in terms of the white blood cell count, proportions of segmented neutrophils and lymphocytes, blood levels of pyruvate, lactate, or alanine, or the normalized urinary excretion levels of pyruvate, lactate, and alanine with creatinine. Three of the study patients had concurrent infections at baseline, including one responder and two non-responders. The HP [1-^13^C]-lactate-to-tC ratio (%) was higher in the infected group than in the noninfected group (mean, 13.0 ± 6.2 *versus* 10.4 ± 6.2; *n* = 3, *p* = 0.775).

**Table 2:** Baseline immune metabolic characteristics between responders and non-responders

Measurement	Responder	Non-responder	*p*-value
Total HP ^13^C signal (tC) (10^12^)	2.3 ± 0.3	2.4 ± 0.1	.725
HP [1-^13^C]-pyruvate-to-tC ratio (%)	91.5 ± 3.5	74.4 ± 1.6	.011
HP [1-^13^C]-lactate-to-tC ratio (%)	3.6 ± 2.5	19.9 ± 2.4	.009
HP [1-^13^C]-alanine-to-tC ratio (%)	0.7 ± 0.2	0.9 ± 0.6	.767
HP [^13^C]-bicarbonate-to-tC ratio (%)	0.6 ± 0.4	0.2 ± 0.0	.334
Spleen volume (10^2^ cm^3^)	1.1 ± 0.0	1.5 ± 0.3	.161
Spleen ADC (10^−3^ mm^2^/s)	1.2 ± 0.0	1.6 ± 0.3	.199
WBC count (10^3^/mL)	7.6 ± 0.4	12.1 ± 5.2	.443
Segmented neutrophil (%)	69.4 ± 0.9	70.4 ± 9.4	.918
Lymphocyte (%)	24.3 ± 1.1	19.3 ± 7.5	.545
Blood pyruvate (dmol/L)	0.2 ± 0.0	0.2 ± 0.0	.897
Blood lactate (dmol/L)	3.1 ± 0.6	2.8 ± 0.8	.812
Blood alanine (dmol/L)	0.7 ± 0.1	0.6 ± 0.2	.956
Urine pyruvate/creatinine (%)	0.7 ± 0.1	0.7 ± 0.1	.954
Urine lactate/creatinine (%)	2 ± 0.4	3.2 ± 0.4	.101
Urine alanine/creatinine (%)	3.4 ± 0.8	4 ± 0.9	.677
Tumor size (cm)	3.9 ± 0.6	5.3 ± 0.8	.240
Tumor ADC (10^−3^ mm^2^/s)	0.8 ± 0.0	0.8 ± 0.0	.599

Data are mean ± standard error. *ADC* Apparent diffusion coefficient, *HP* Hyperpolarized, *WBC* White blood cell

**Fig. 5:**
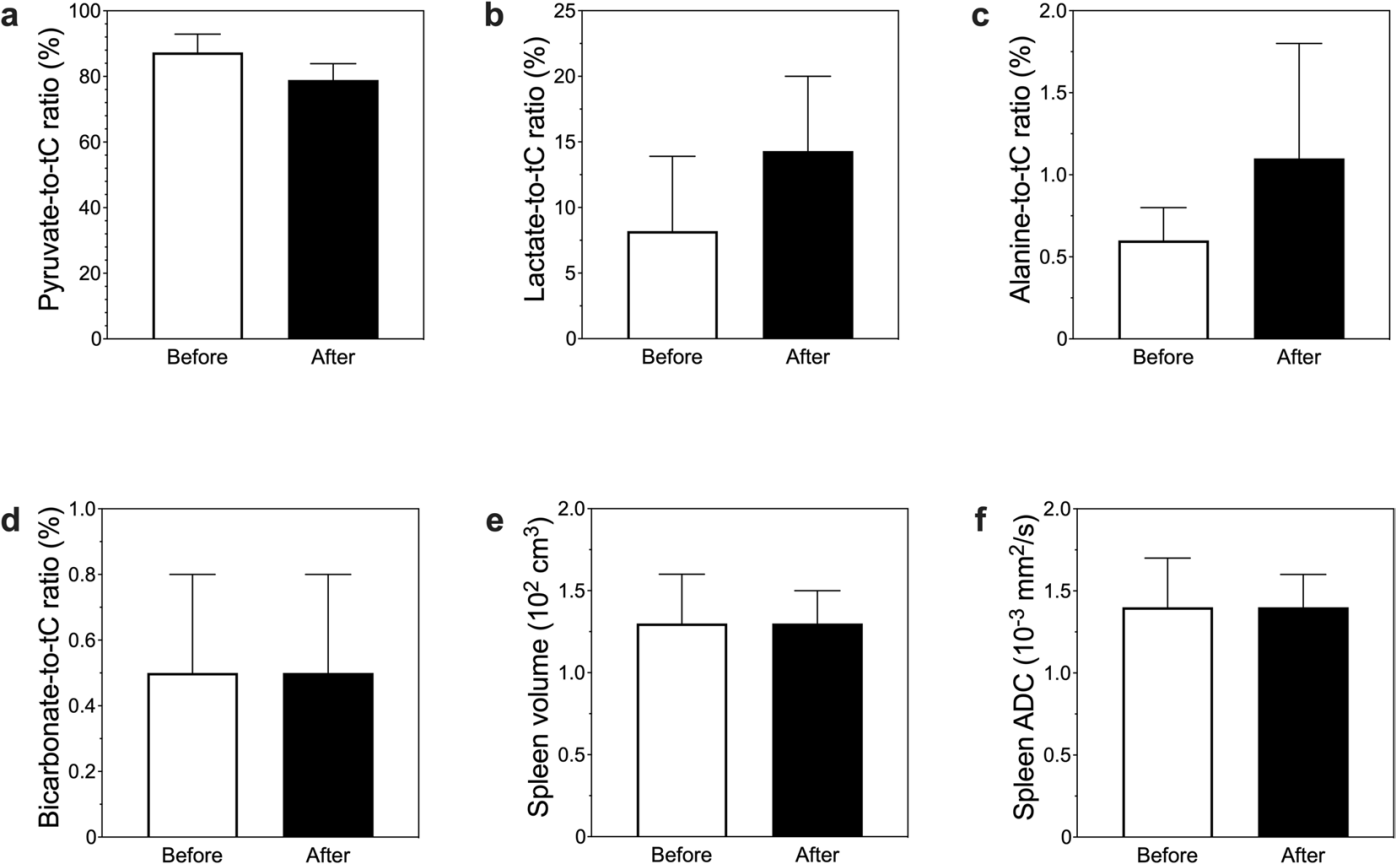
Hyperpolarized MR spectroscopy and imaging of the spleen before and after radiotherapy. The splenic hyperpolarized (HP) [1-^13^C]-lactate-to-tC ratio (%) revealed a 1.7-fold increase (**b**), and the splenic [1-^13^C]-alanine-to-tC ratio (%) revealed a 1.8-fold increase (**c**) after radiotherapy, albeit without statistical significance. There were no significant differences observed in the HP [^13^C]-pyruvate-to-tC ratio (**a**) and HP [.^13^C]-bicarbonate-to-tC ratio (**d**) as well as the splenic volume (**e**) or apparent diffusion coefficient (ADC) values (**f**)

The blood leukocyte differential count 2 weeks after treatment revealed an increased proportion of segmented neutrophils from 65.9% ± 4.1% to 81.0% ± 2.8% (*n* = 4, *p* = 0.013), indicating enhanced immune activity. The splenic [1-^13^C]-lactate signals (lactate-to-tC ratio [%]) revealed a 1.7-fold increase (mean, 8.2 ± 5.7 *versus* 14.3 ± 5.7; *n* = 4, *p* = 0.415), and the splenic [1-^13^C]-alanine-to-tC ratio (%) revealed a 1.8-fold increase after radiotherapy (mean, 0.6 ± 0.2 *versus* 1.1 ± 0.7; *n* = 4, *p* = 0.482, Fig. 5). In the responder group, a trend of increasing splenic HP [1-^13^C]-lactate-tC ratio (%) was observed before and after treatment (mean, 8.2 ± 5.7 *versus* 14.3 ± 5.7; *n* = 3, *p* = 0.415). The tumor ADC values showed a significant increase between before and after radiotherapy for responders (mean, 0.8 ± 0.0 *versus* 1.0 ± 0.0 × 10^-3^ mm^2^/s; *n* = 3, *p* = 0.022) and non-responders (mean, 0.8 ± 0.0 *versus* 0.9 ± 0.0 × 10^-3^ mm^2^/s; *n* = 3, *p* = 0.046).

Two-dimensional images of the left upper abdomen were obtained in all participants to confirm the source of HP ^13^C signals. An example is shown in Fig. 3. [1-^13^C]-pyruvate, [1-^13^C]-lactate, [1-^13^C]-alanine, and [^13^C]-bicarbonate maps were obtained, which confirmed that the HP signals were primarily from the spleen, but not from the adjacent tissues such as muscle, fat, stomach, aorta or splenic artery. Patients showed no evidence of bacteremia, psoriasis, or autoimmune diseases and tested negative for coronavirus disease (COVID-19) in the polymerase chain reaction test. No adverse events were detected during the follow-up period, and there were no significant changes in vital signs, hematology, electrocardiography, or other safety variables, indicating unremarkable data on serum biochemistry variables and post-dosing changes.

## Discussion

This study utilized HP [1-^13^C]-pyruvate MRS to evaluate pyruvate metabolism in the human spleen and identified significantly lower splenic lactate levels in responders as compared with non-responders, providing a potential method for identifying candidates for radiotherapy in cervical cancer. We found the baseline splenic HP [1-^13^C]-lactate-to-tC ratio was 5.6-fold significantly lower in the responders than in the non-responders, and the splenic [1-^13^C]-lactate-to-tC ratio revealed a 1.7-fold increase following radiotherapy.

The primary cause of alterations in splenic lactate resulting from HP ^13^C pyruvate appears to stem from immunometabolism rather than being a consequence of decreased perfusion. This is supported by the absence of variations in splenic volume between responders and non-responders as well as the lack of detectable ^13^C signal originating from the nearby aorta or splenic artery. The standard-of-care diffusion-weighted MRI did not indicate such correlation between the immune potential and the splenic cellularity.

Our results are in line with previous reports that have demonstrated an association between baseline splenic [^18^F]-FDG–PET/CT spleen-to-liver standard uptake value > 0.94 and unfavorable oncological outcomes as well as a negative effect on pathological complete response in cervical cancer patients [16]. The results indicate that spleen metabolism may serve as a potential marker for an individual's immune potential and that radiation therapy has the potential to trigger the abscopal effect, which is a systemic antitumor response involving immunological mechanisms such as metabolic reprogramming [4] and lymphocyte activation [3]. Additionally, pretreatment [^18^F]-FDG–PET/CT imaging has been used to assess systemic inflammation through the metabolism of lymphoid organs such as the bone marrow and spleen [17], although caution is warranted as increased FDG uptake in the spleen has been associated with bacteremia, subclinical atherosclerosis in psoriasis [18], and febrile autoimmune disease [19].

In the present study, splenic lactate-to-tC ratio increased after treatment, suggesting potential immune activation. Moreover, our findings suggested a potential trend towards an increase in the alanine to total carbon ratio in the responder group. This suggests a possible inclination towards an alternative metabolic pathway for pyruvate utilization in the spleen post-treatment, possibly shifting from lactate to alanine, albeit without statistical significance. Measuring splenic immune potential may have potential applications beyond radiotherapy and could also be used in cancer immunotherapy. One study compared the predictive value of inflammatory biomarkers from pretreatment peripheral blood and [^18^F]-FDG–PET/CT in estimating outcomes in patients with non-small-cell lung cancer treated with first-line immunotherapy or chemotherapy [20], which may be useful in predicting progression-free and overall survival. Another study reported that spleen-to-liver ratios of > 1.1 before ipilimumab treatment for advanced melanoma were associated with poor outcomes [21]. To this end, the application of HP [1-^13^C]-pyruvate MRS to assess metabolism in the human spleen has potential applications in the study of immune metabolism, such as examining the proton therapy abscopal effect, activating immunotherapy, adverse events from immunotherapy, transplant rejection, autoimmune diseases, and monitoring the long-term effects of COVID-19.

This study was subject to certain limitations. Firstly, patient recruitment for the clinical trial was challenging due to a global shortage of sterile fluid paths during the COVID-19 pandemic, making it difficult to expand the case number. Additionally, preparation of the HP [1-^13^C]-pyruvate dose encountered unexpected technical failures, which could limit routine clinical use. Measuring metabolism using ^13^C HP MRI in the primary tumor was not able to be also undertaken at the same time, due to the scan field constraint limited by the only available surface coil for the present HP ^13^C study. Secondly, while our study did not analyze the spatial heterogeneity of the spleen, it should be noted that our patients did not have known lymphoma or focal splenic lesions. Thirdly, hydronephrosis is not uncommon in patients with locally advanced cervical cancer. Additional cases are needed to investigate the confounding effect of infections. Lastly, a longer follow-up period is necessary to establish the relationship between our initial findings and patients’ long-term survival.

In conclusion, this exploratory study revealed the feasibility of HP [1-^13^C]-pyruvate MRS of the spleen for evaluating baseline immune potential, which was associated with clinical outcomes of cervical cancer after radiotherapy. This technique opens up new avenues for noninvasive assessment of immune characteristics and could be useful in selecting candidates for future immune-related clinical trials.

### Supplementary Information

**Additional file 1.**

## Data Availability

All data were generated by the authors and are available upon request to the corresponding authors of this study.

## Abbreviations

ADC: Apparent diffusion coefficient
COVID-19: Coronavirus disease 2019
FDG: 2-Deoxy-2-[^18^F]-fluorodeoxyglucose
FIGO: International Federation of Gynecology and Obstetrics
HP: Hyperpolarized
MRI: Magnetic resonance imaging
MRS: Magnetic resonance spectroscopy
PET/CT: Positron emission tomography/Computed tomography
tC: Total carbon
