# Supplementary material for: Hyperpolarized [1-13C]-pyruvate MRS evaluates immune potential and predicts response to radiotherapy in cervical cancer
Source: Eur Radiol Exp. 2024 Apr 10;8:46. doi: 10.1186/s41747-024-00445-1 (PMC11003947; doi:10.1186/s41747-024-00445-1)
Supplement: Supplementary file 1 — Additional file 1. [file 41747_2024_445_MOESM1_ESM.docx]

**Hyperpolarized [1-^13^C]-pyruvate MRS evaluates immune potential and predicts response to radiotherapy in cervical cancer**

**ELECTRONIC SUPPLEMENTARY MATERIAL**

# **Study Visits and Protocol**

Following at least 4 hours fast before the study, participants arrived at the clinical Magnetic Resonance Center at approximately 11:30 AM. After confirming informed consent, vital signs, including pulse, breath, blood pressure and body temperature, were obtained. One intravenous line, 20 gauge, was placed in the left or right median cubital vein. After completion of the study, participants returned to a procedure room for vital signs, removal of the intravenous line, and observation for 30 minutes. Total duration of the study visit was approximately 2 hours from time of arrival to discharge.

# **Production of HP [1-^13^C]-pyruvate**

The sterile fluid path pharmacy kits (SFP, GE Research Circle Technology Inc) were filled in the laminar flow located in the clean room: 1.46 ± 0.01 g of 14 M [1–13C]pyruvic acid (PA, API grade, Sigma-Aldrich W6578) containing 12.5 mM of electron paramagnetic agent (EPA; AH111501, Syncom) were filled in the sample vial and sealed by laser welding (Leister Technologies). The sample vial was loaded in the SPINlab (GE Research Circle Technology Inc), and then polarized under the environment of 0.7 to 0.8 K and irradiated at 139.9 GHz by microwave for 3–6 hours. The solid mixture of PA/EPA was dissolved in 37.45 ± 0.05 g of superheated sterile water, subsequently passed through the EPA filter to remove excessive excipient and buffered with the mixture of 18.15 ± 0.05 g of sterile water and 18.25 ± 0.05 g of neutralization medium (600 mM NaOH/333 mM Trizma base/333 mg/L Na2EDTA), and then filtered by a 0.2 μm terminal filter (Saint-Gobain) into the administration syringe (MedRad, Bayer Healthcare). Quality control were automatically performed in the QC system (GE Research Circle Technology Inc), the release criteria were as follows: pyruvate concentration 200 to 280 mM, EPA residual concentration < 5 μM, pH 5 to 9, temperature 25 °C to 37 °C and polarization level not less than 10 %.

The concentration of pyruvate and polarization level measured immediately after the dissolution was 228.2 ± 10.6 mM (mean ± SD, n = 10) and 27.7 ± 7.0 %, respectively. Concentration of remaining EPA in the final HP pyruvate solution was 2.1 ± 0.7 μM. The dissolution to injection time and scanning time were 68.8 ± 3.5 s and 98.9 ± 3.4 s, respectively. The integrity of the filter was immediately tested after wetting it by performing 50 PSI bubble-point testing. The final injectate was administered at 0.43 mL/kg at 5 mL/s, followed by flushing with 20 mL of normal saline. HP [1-^13^C]-pyruvate injection was well tolerated by all participants. None of the participants reported an unusual taste or a sensation of flushing. All participants were monitored for 1 h after the HP injection, and no adverse effects were noted. No significant changes were observed in the vital signs or ECG findings.

# **MRI Exams**

All participants laid down in supine, with of the coil center line was placed closely to spleen. All MRI studies were performed on a 3-T 750 W Discovery MRI scanner (GE Healthcare). The study used a flex RF coil that consisted of ^1^H linear transmit/receive (mean diameter = 12.1 cm), 13C linear transmit (mean diameter = 10.9 cm). The dimensions of coil are length 23.9 cm, width 14.0 cm, and height 0.25 cm, respectively. The spleen location relative to the RF coil was confirmed using a single-shot fast spin echo (field-of-view [FOV] = 45 cm × 45 cm, slice thickness = 10 mm, spacing = 5 mm, echo time [TE] = 80 ms, repetition time [TR] = 900 ms, scan time = 20 s). Time-resolved free-induction-decay (FID) data were acquired using a slice-selective pulse-and-acquire sequence for ^13^C MR spectroscopy (slice thickness = 3 cm, flip angle = 10°, TR = 2 s, spectral width = 5,000 Hz, spectral point = 4096, scan time = 28 s). A metabolite-selective multi-echo spiral imaging sequence (slice thickness = 3 cm, flip angle = 10° for pyruvate and 20° for products, total scan time = 1400 ms) was used for ^13^C MRI to image [1-^13^C]-pyruvate, [1-^13^C]-lactate, [1-^13^C]-alanine, and [^13^C]-bicarbonate sequentially using a spectral–spatial RF pulse that selectively excites the labeled metabolites. [1-^13^C]-Pyruvate-hydrate, which is a metabolically inactive molecule, was not imaged because its distribution is similar to that of [1-^13^C]-pyruvate due to their instant equilibrium. The generic magnetic resonance spectrum imaging (MRSI) (slice thickness = 2 cm, flip angle = 10°, TR = 0.0832 s, spectral width = 5,000 Hz, spectral point = 352, grid size 8 (row) x 8 (column), scan time = 5.32 s) was implemented. The MRSI sequence was used only in the first two cases due to limited information.

Details of MR acquisition protocols for morphological T2-weighted, and DW MR imaging (b = 0, 1000 sec/mm2) are following: (DW: single-shot echo-planar technique with fat suppression; TR/TE, 6800/71.2; average, 3; b-value = 0 and 1000 sec/mm2, section thickness, 5 mm; gap, 1 mm; matrix, 256 x 256; FOV, 32 cm; T2-weighted: 1529.2/132; matrix, 256 x 192; FOV, 20 cm). This study was performed during minimal breathing.

# **Data Reconstruction and Analysis**

Acquired HP ^13^C data were reconstructed using MATLAB (Mathworks, version R2020b). FID was apodized with a 3-Hz Hanning filter at each timepoint and Fourier transformed. The complex spectra were combined in absorption mode after 0th and 1st order phase corrections, then divided by Equation A1, which is the coefficient to compensate differences in QC parameters such as liquid-state polarization level (%) and pyruvate concentration (mM) and experimental parameters such as transfer time from dissolution to injection(s). *In vitro* T1 for pyruvate (78 s) was used to calculate the polarization loss during the transfer time ([10](#_ENREF_10)).

$$\frac{{\text{[}\text{pyruvate}\text{]}}_{\text{QC}}}{250}\boldsymbol{\cdot}\frac{\text{polarization}_{\text{QC}}}{100}\boldsymbol{\cdot}e^{-\frac{\boldsymbol{T}_{\boldsymbol{transfer}}}{\boldsymbol{T}_{\boldsymbol{1,pyruvate}}}} \mathbf{-}\left( A1 \right)$$

For 13C imaging, the k-space data was gridded to the Cartesian coordinate, zero-filled by a factor of 4, and applied by a 2D inverse Fourier transform.

# **^1^H Nuclear Magnetic Resonance (NMR) Spectroscopy**

A Bruker Advance 600-MHz spectrometer equipped with a 5-mm CPTCI 1H cryoprobe was used to perform NMR spectroscopy **(**Bruker GmbH, Karlsruhe, Germany**)**. The Bruker pulse sequence *noesygppr1d* was employed, with continuous-wave irradiation at the water frequency using an RF strength of 25 Hz, a relaxation delay of 4 s, and a mixing time of 10 ms. A magnetic field z-gradient was applied for 1 ms, and the acquisition time was 2.7 s, with a spectral window of 20 ppm and a receiver gain of 90.5. The overall TR of the acquisition sequence was 6.7 s, and the temperature was kept at 300 K during the experiment. Data processing was performed with a line broadening setting of 0.3 Hz, zero-filling by a factor of 2 to produce 128k Fourier domain points. The NMR spectra obtained were manually phased, baseline corrected, and referenced to the chemical shift of TSP (δ 0.0 ppm) using TopSpin 3.2 software **(**Bruker BioSpin, Rheinstetten, Germany**)**.

**NMR Data Processing and Analysis**

To identify the metabolites in blood and urine, the full-resolution NMR data were analyzed using Chenomx NMR Suite 8.1 professional software **(**Chenomx Inc., Edmonton, AB, Canada**)**. The NMR spectra were aligned and normalized to the TSP spectral area to calculate the concentration of each metabolite. Regions in the dataset containing residual water (δ 4.825–4.725 ppm) were excluded to prevent spectral interference. A standard two-dimensional NMR experiment was conducted on blood and urine samples, and metabolites were identified by comparing them with reference spectra from the Human Metabolome Database (<https://hmdb.ca>).

# **Radiotherapy protocol**

Patients underwent external beam radiotherapy for 5 days each week, with one fraction per day at a dose of 1.8 Gy per fraction. A conventional four-field box technique was used to deliver a dose of 45 Gy of large-field radiation to the entire pelvis. Patients with parametrial extension were administered parametrial booster doses (5.4−12.6 Gy) using parallel-opposed anterior/posterior fields with a 4-cm-wide midline block. External beam doses to the lower pelvis were increased to 50−54 Gy without a central block if followed by brachytherapy or to 68−72 Gy in patients with lower vaginal tumor extension or who presented with persistent bulky tumors after receiving 45 Gy of irradiation. Intracavitary high-dose-rate ^192^iridium brachytherapy was administered at 4.3 Gy per fraction for six fractions, with two fractions per week. During radiotherapy, chemotherapy was given as a weekly intravenous infusion of cisplatin (40 mg/m2) for up to six cycles. Dose reductions or withholding of chemotherapy were considered if hematologic toxicity occurred (granulocyte count <1,500/mL or platelet count <100,000/mL). Patients who completed their radiotherapy course were not required to complete all six cycles of chemotherapy.
